# High-purity magnesium screws modulate macrophage polarization during the tendon–bone healing process in the anterior cruciate ligament reconstruction rabbit model

**DOI:** 10.1093/rb/rbac067

**Published:** 2022-10-10

**Authors:** PengFei Cheng, ZhenJun Weng, Musha Hamushan, Weijie Cai, Yubo Zhang, Zun Ren, Yunchu Sun, XiaoNong Zhang, Hao Shen, Pei Han

**Affiliations:** Department of Orthopedic Surgery, Shanghai Jiao Tong University Affiliated Sixth People's Hospital, Yishan Rd 600, Shanghai 200233, China; Department of Orthopedic Surgery, Shanghai Jiao Tong University Affiliated Sixth People's Hospital, Yishan Rd 600, Shanghai 200233, China; Department of Orthopedic Surgery, Shanghai Jiao Tong University Affiliated Sixth People's Hospital, Yishan Rd 600, Shanghai 200233, China; Department of Orthopedic Surgery, Shanghai Jiao Tong University Affiliated Sixth People's Hospital, Yishan Rd 600, Shanghai 200233, China; Department of Orthopedic Surgery, Shanghai Jiao Tong University Affiliated Sixth People's Hospital, Yishan Rd 600, Shanghai 200233, China; Department of Orthopedic Surgery, Shanghai Jiao Tong University Affiliated Sixth People's Hospital, Yishan Rd 600, Shanghai 200233, China; Department of Orthopedic Surgery, Shanghai Jiao Tong University Affiliated Sixth People's Hospital, Yishan Rd 600, Shanghai 200233, China; State Key Laboratory of Metal Matrix Composites, School of Materials Science and Engineering, Shanghai Jiao Tong University, Shanghai, 200240, China; Suzhou Origin Medical Technology Co. Ltd, Suzhou, 215513, China; Department of Orthopedic Surgery, Shanghai Jiao Tong University Affiliated Sixth People's Hospital, Yishan Rd 600, Shanghai 200233, China; Department of Orthopedic Surgery, Shanghai Jiao Tong University Affiliated Sixth People's Hospital, Yishan Rd 600, Shanghai 200233, China

**Keywords:** high-purity magnesium, tendon–bone healing, anterior cruciate ligament reconstruction, macrophage polarization

## Abstract

Magnesium (Mg) screws perform clinical potential in anterior cruciate ligament (ACL) reconstruction, and promote fibrocartilaginous entheses regeneration at the femoral entrance. We aim to prove that high-purity Magnesium (HP Mg) screws modulate macrophage polarization in fibrocartilage interface regeneration both *in vitro* and *in vivo*. HP Mg extracts performed good cytocompatibility and significantly promoted M2 macrophage polarization in the flow cytometry and ELISA assays. M2 macrophages stimulated fibrochondrocyte differentiation of co-cultured hBMSCs, and HP Mg extracts had synergistic effect on the process. Then we applied HP Mg screws, with Ti screws as control, in the ACL reconstruction rabbit model. In the histological and immunofluorescence analysis, HP Mg screws inhibited M1 polarization at 2 weeks and highly promoted M2 polarization at 2 and 4 weeks at the tendon–bone interface. Furthermore, regeneration of fibrocartilaginous entheses, rather than the fibrovascular scar interface, was detected in the HP Mg group at 12 weeks. For further mechanism study via RNA-seq detection and WB assays, we found that AKT1 was highly activated in M2 polarization, and HP Mg could stimulate AKT1 expression, rather than AKT2, in the early phase of tendon–bone healing. Our study elucidated macrophage polarization during tendon–bone healing process and emphasized HP Mg on M2 polarization and fibrocartilage interface regeneration via the selective activation of AKT1 and PI3K/AKT pathway.

## Graphical Abstract

**Figure:**
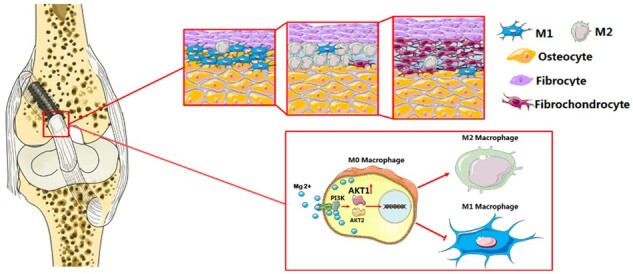

## Introduction

Anterior cruciate ligament (ACL) reconstruction with autologous tendon graft fixed by the interference screws to the bone tunnel is a widely performed procedure for the stability and function of the knee joint. Approximately 4 million patients suffer from ACL injuries worldwide and about half of them undergo ACL reconstruction [1]. Although clinical outcomes of ACL reconstruction are generally favorable, around 10% autograft repair cases and 25% allograft repair cases may face treatment failure and require a second revision [2]. Among various contributors to poor prognosis, such as traumatic reinjury, technical errors, and iatrogenic causes, the insecure tendon–bone healing is the most frequently observed in clinical practice [3]. It often causes graft slippage in the tunnel, increasing joint laxity and accelerating knee osteoarthritis [4]. Hence, it is exigent for both basic and clinical researchers to explore new strategies specifically targeting the tendon–bone healing process.

Considering their splendid biocompatibility, desirable mechanical properties similar to the bone, and adequate corrosion resistance within the bone tunnels, Magnesium (Mg)-based biomaterials have been intensively studied and applied to the interference screws [5–7]. Meanwhile, the development of mechanical properties of Mg-based interference screws has met the clinical demands in ACL reconstruction. Thus, a series of interference screws, made of the novel Mg-based materials with optimal content, have been introduced to counter the commercialized titanium (Ti) alloy screws and the polylactic acid screws [8]. Our previous study applied the high-purity Magnesium (HP Mg) interference screws in a rabbit model of ACL reconstruction, in which the regeneration of fibrocartilaginous entheses at the intra-articular site was significantly improved [9, 10]. Other studies also suggested that the application of HP Mg and Mg–6Zn–0.5Sr screws in ACL reconstruction could improve tendon graft osteointegration within the bone tunnel and mineralization at the tendon–bone interface [11, 12]. Therefore, Mg-based interference screws have shown great potential in the enhancement of bone-graft healing quality. However, how Mg-based materials mechanistically modulate the biological ACL reconstruction process remains unclear.

Macrophage activity plays an essential role in the process of tendon–bone healing. Reconstruction of the ACL begins with an early influx of inflammatory cells and subsequent graft necrosis, followed by the formation of fibrovascular interface tissue (the revascularization process) and, finally ends with the intra-articular and the intra-tunnel remodeling of graft tissue [3]. Exposed to the microenvironment of the tendon–bone healing process, local and blood-derived macrophages are phenotypically polarized into classically activated macrophages (M1 type) and alternatively activated macrophages (M2 type) [13]. M1 macrophages promote the Th1 response and produce key pro-inflammatory cytokines, such as TNF-α and IL-6 [14]. In contrast, M2 macrophages promote the Th2 response during the phase of tissue remodeling, downregulate inflammatory cytokines and produce anti-inflammatory factors including Arg1 and TGF-β [15]. Recent evidence suggests that macrophage polarization, especially M2 polarization, could contribute to the secretion of growth factors and anti-inflammatory cytokines responsible for fibroblast mitogenesis and proliferation, as well as the synthesis of extracellular matrix and collagen, during the tendon–bone healing process [16, 17]. Therefore, strategies to modulate macrophage polarization could be used to reveal the underlying mechanisms of tendon–bone healing and pave the way for related clinical researches and applications.

It is still controversial how the Mg-based materials impact the inflammatory response and related mechanisms. S. Schumacher et.al reported that pure Mg in degrading process could increase IL-8 secretions and elicit proinflammatory reactions in the primary porcine nasal epithelial cells [18]. On the other hand, Sugimoto *et al.* found reduced maternal TNF-α and IL-6 production in patients who received MgSO_4_ therapy. Also, an *in vitro* study where neonatal monocytes were exposed to a clinically effective MgSO_4_ concentration showed a similar anti-inflammatory pattern of Mg [19]. Additionally, our previous animal studies suggested that Mg screws could inhibit the inflammatory reaction at the tendon–bone interface and enhance collagen regeneration of tendon graft in the early ligamentization phase [10]. However, whether Mg-based screws could influence the tendon–bone healing process via coordinating macrophage polarization and the inflammatory responses remains a research gap. Also, the immunoregulatory effects of Mg products in the process of ACL reconstruction and the underlying mechanism need to be elucidated.

In this study, we aim to investigate whether Mg-based materials are capable of modulating macrophage polarization during the inflammatory phase and reconstruction phase of tendon-to-bone healing *in vitro* and *in vivo*, with a particular emphasis on molecular signaling pathways involved. Specifically, the effect of HP Mg on immunomodulation during fibrocartilaginous entheses regeneration was evaluated through bioinformatics analysis and other methods.

## Materials and methods

### Interference screws preparation

**Figure 1.**
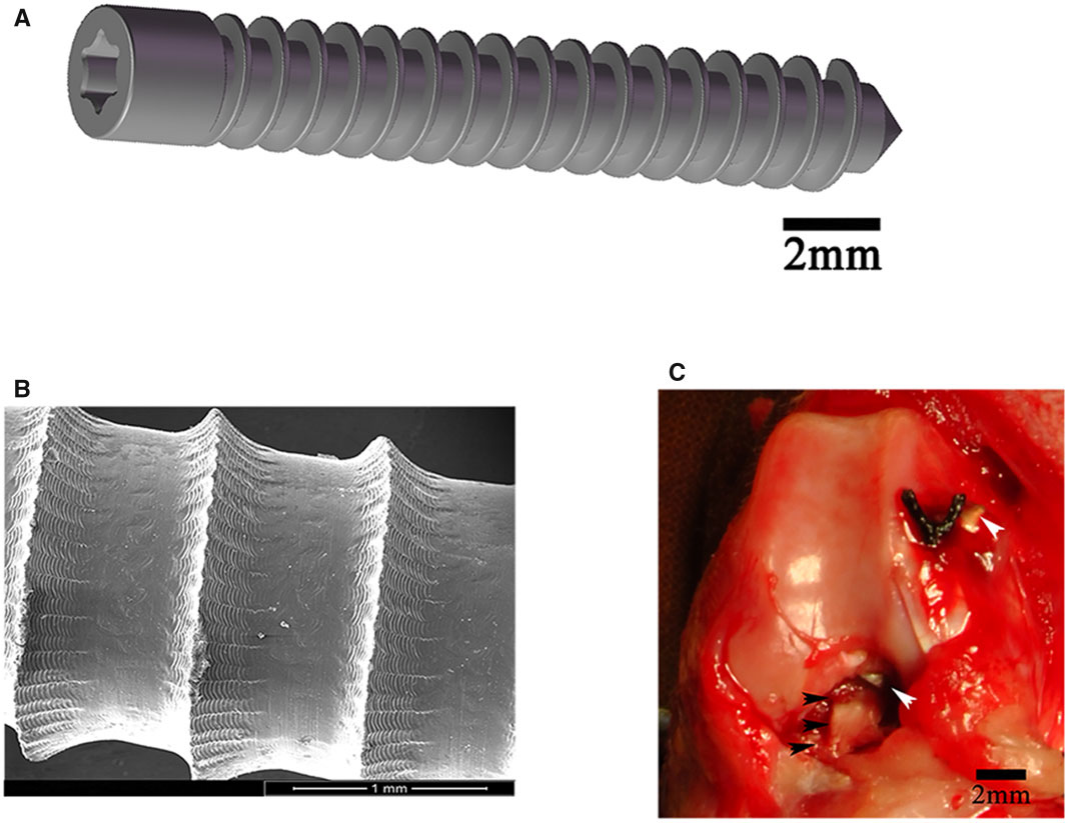
(**A**) Diagrammatic drawing of high-purity magnesium (HP Mg) interference screws (scale bar means 2 mm). (**B**) Surface morphology of HP Mg screws (scale bar means 1 mm). (**C**) Anterior cruciate ligament (ACL) reconstruction with semitendinosus (black arrowheads) fixed by HP Mg screws (white arrowheads) into the femoral tunnel (scale bar means 2 mm).

As suggested in a previous report, the ultimate tensile strength, the yield strength, and the elongation at break of HP Mg (99.99 wt.%) were about 199.1, 148.5 MPa, and 8.1% after the extruding process [20]. The HP Mg interference screws (outer diameter and inner diameter of 2.7 and 2.1 mm, respectively) were designed according to the rabbit knee joint anatomy, produced and assembled before sterilization using 29 kGy of Co-60 radiation. Morphologically resemble Ti screws were manufactured concurrently as the research control. All screws used in the current study showed clear threads and a smooth surface without irregular or isolate holes (Fig. 1A and B).

HP Mg extracts were prepared after immersion of HP Mg screws in DMEM supplemented with 10% FBS, 100 U/ml penicillin, 100 mg/ml streptomycin and 0.1 mg/ml sodium pyruvate at 37°C in a humidified atmosphere of 5% CO_2_ for 24 h. HP Mg extracts were measured for Mg ion concentration and pH value, respectively.

### *In vitro* cell experiment

#### Macrophage polarization and cell intervention

The murine macrophage-like cell line RAW 264.7 was maintained in DMEM supplemented with 10% FBS, 100 U/ml penicillin, 100 mg/ml streptomycin and 0.1 mg/ml sodium pyruvate and then treated with different inducers to establish M1 and M2 phenotypes as follows. The M1 phenotype was induced by culturing matured macrophages for 2 days in the presence of 1 × 10^3^ U/ml recombinant human IFN-γ (Abcam). In day 2, 10 ng/ml LPS-EB Ultrapure (InvivoGen, USA) was added into the medium to induce an M1 phenotype. M2 macrophages were analogously generated by adding 10 ng/ml human IL-4 (ImmunoTools) and 10 ng/ml IL-10 (ImmunoTools) to the culture medium.

Next, induced M1 macrophages and M2 macrophages were treated with HP Mg extracts (M1-Mg, M2-Mg) for 24, 48 or 72 h separately, while purely cultured M1/M2 macrophages served as the control groups (M1-Con, M2-Con). Conditioned medium (CM) of polarized macrophages in each group were harvested after treatment for 72 h. The supernatant CM in each group (M1-Mg group, M1-Con group, M2-Mg group and M2-Con group) were harvested after repeated centrifugation.

#### Co-culture of hBMSCs and polarized macrophages

Human bone marrow-derived mesenchymal stem cells (hBMSCs) were supplied by the Shanghai Jiao Tong University Affiliated Sixth People's Hospital, according to reported method. hBMSCs were incubated in the α-MEM culture medium with 10% FBS, 100 U/ml penicillin and 100 mg/ml streptomycin at 37°C in a humidified atmosphere of 5% CO_2_ for 24 h. After 24 h of incubation, hBMSCs were co-cultured with polarized macrophages-CM with or without HP Mg extracts treatment (M1-Mg group, M1-Con group, M2-Mg group and M2-Con group), with the control group treated with α-MEM with 10% FBS, 100 U/ml penicillin and 100 mg/ml streptomycin. The hBMSCs were harvested for RT-PCR assay after co-culturing for 3 days, or 14 days for glycosaminoglycan (GAG) quantifications.

#### CCK-8 assay

CCK-8 assay was performed using a commercial kit (Dojindo Molecular Technology, Japan) to evaluate cell proliferation. According to the manufacturer’s instructions, cell suspension (100 μl/well) was inoculated in a 96-well plate and mixed with 10 μl CCK-8 solution. The plate was then incubated in the humidified environment (37°C, 5% CO_2_) for up to 4 h before being subjected to the microplate reader for absorbance measurement at 450 nm.

#### Cell invasion assay

To evaluate cell invasion, macrophages (1 × 10^5^/ml, in DMEM supplemented with 1% FBS) were plated into the upper Transwell chamber (8-µm pores Transwell inserts, BD BioCoatTM). The lower chamber was filled with serum-free 0.1% BSA-DMEM. After 24 h, uninvaded cells were removed from the upper surface of the Transwell membrane, and invaded cells in the lower chamber were fixed and stained for further observation and counting.

#### Colony formation assay

RAW264.7 cells were seeded in triplicate in 48-well plates at the density of 3 × 10^3^ cells/well and cultured for 4 days with or without HP Mg extracts. At the end of Day 4, the cells were fixed and stained with 40, 6-diamidino-2-phenylindole (DAPI). Colonies or areas with more than 50 nuclei were counted.

#### Flow cytometry assay

Flow cytometry was applied to analyze the expression of M1 specific marker CD11c and M2 specific marker CD206. Cellquest Pro software (Becton Dickinson) and FlowJo software version 9.4.0 (Tree Star, San Carlos, CA, USA) were utilized for data processing and results were presented as mean fluorescent intensity (MFI).

#### Enzyme-linked immunosorbent assay

Inflammatory cytokines (IL-6, TNF-α) and tissues repair cytokines (Arg1, TGF- β) secreted into the culture supernatant by macrophages were evaluated using commercial enzyme-linked immunosorbent assay (ELISA) kits (Abcam, Cambridge, CA, USA) according to the manufacturer's instructions.

#### RNA-Seq and bio-informatic analysis

Total RNA was extracted from M2-Con or M2-Mg using a RNeasy Mini Kit (Qiagen, Hilden, Germany). An MGI Easy™ mRNA Library Prep Kit (BGI Inc., Wuhan, China) was used for library preparation before cluster generation and sequencing on the GISEQ-500 system (BGI). Detailed sequencing settings were described in our previous work [21]. All the reference genes were from Rattus norvegicus-UCSC_rn6 (ftp://hgdown-load.cse.ucsc.edu/goldenPath/rn6) and the KEGG pathway enrichment analysis was performed using the Database for Annotation, Visualization and Integrated Discovery (DAVID).

#### Real time-polymerase chain reaction assay

The RT-PCR analysis was done based on the LightCycler1 Nano System (Roche Diagnostics, Mannheim, Germany). Briefly, the mixture of LightCycler FastStart DNA Master SYBR Green (Roche Diagnostics, Mannheim, Germany), PCR-grade water, cDNA and primers in a total volume of 20 μl was subjected to the following program for transcript amplification: 95°C for 600 s, 20 s at 95°C, 20 s at melting temperature (Tm), and 20 s at 72°C, for 45 cycles. Primers used in the current study were presented in Table 1. The 2^−ΔΔCt^ algorithm was used to calculate the expression of mRNA.

**Table 1.** Primers for the RAW 264.7 and hBMSC in the RT-qPCR analysis

Name	Primer	Sequence
GAPDH (RAW 264.7)	Forward	5′-ATGGGTGTGAACCACGAGA-3′
	Reverse	5′-CAGGGATGATGTTCTGGGCA-3′
Akt1 (RAW 264.7)	Forward	5′-AATGCACGGCGATTACACTC-3′
	Reverse	5′-GGACACTGGGTAGAGCAACT-3′
Akt2 (RAW 264.7)	Forward	5′-CTGCCCTTCTACAACCAGGA-3′
	Reverse	5′-CATACACATCCTGCCACACG-3′
18s (hBMSCs)	Forward	5′-GTTCTTAGTTGGTGGAGCGATTT-3′
	Reverse	5′-CGGACATCTAAGGGCATCACA-3′
Aggrecan (hBMSC)	Forward	5′-GGCTGCTGTCCCCGTAGAAGA-3′
	Reverse	5′-GGGAGGCCAAGTAGGAAGGAT-3′
COL2A1 (hBMSC)	Forward	5′-GCTCCCAGAACATCACCTACC-3′
	Reverse	5′-TGAACCTGCTATTG CCCTCT-3′

#### Western blot assays

Proteins were extracted from tissues or cell lysates using a protein extraction kit (Sigma, USA) or RIPA lysis buffer (Beyotime Biotechnology, Shanghai, China). BCA protein assay kit (Beyotime, Shanghai, China) was used to determine the protein concentration in each sample. Equal amounts of proteins were mixed with the loading buffer and subjected to SDS-PAGE, and the proteins were then transferred onto Hybond ECL membranes (Amersham, Buckinghamshire, UK). The membranes were blocked in 5% non-fat milk and incubated with primary antibodies (anti-AKT1, Novus or anti-AKT2, Abcam) at 4°C overnight. The membranes were probed with HRP-labeled secondary antibodies and the protein bands were visualized using an enhanced chemiluminescence system (Kodak, Rochester, NY, USA). Densitometric quantification for all bands was analyzed with Image Pro-plus 6.0 (Media Cybernetics, USA).

#### GAG assay

The GAG concentration of hBMSCs was assayed according to a previously published method [22]. The hBMSCs were washed, digested and then centrifuged. After repeated freezing and thawing, cells were collected and reacted with 1,9-dimethylmethylene blue (DMMB) dye. The concentration of the GAG-DMMB complexes was determined spectrophotometrically at 540 and 595 nm, using a standard curve generated by chondroitin-6-sulfate. The corresponding DNA content was determined using the PicoGreen dsDNA assay (Molecular Probes). The relative GAG content was then normalized to the DNA content.

### Preliminary animal study

#### Animal model and study design

Thirty-six male New Zealand white rabbits (aged 4–6 months; weight 2.5–3 kg) were randomly assigned into two groups for ACL reconstruction with semitendinosus. Specifically, the rabbit knee joint was exposed via a medial parapatellar incision under general anesthesia. After complete ACL transection, the semi-tendinous tendon was harvested. In the position of 45° flexion, a 2.1-mm-wide bone tunnel was created from the tibia to the femoral condyle via the footprint of the original ACL. After countersinking the bone tunnel with a 2.7-mm drill bit, we pulled the semi-tendinous tendon out and fixed it to the femoral tunnel with HP Mg or Ti screws. The tendon graft was fixed to the neighboring periosteum with a suture (Fig. 1C). After modeling, anti-infection treatment (amoxicillin, 15 mg/kg body weight, per day) was offered to all rabbits for 3 days.

#### Functional evaluation

The range of motion (ROM) of the knee joint was measured with a goniometer at different time points post-operation, indicating the functional recovery of the rabbit. The femur-ACL graft-tibia complex sample, with a femur length of 60 mm and a tibia length of 60 mm, was harvested 2, 4 and 12 weeks after modeling, and then fixed in 4% neutral buffered formalin before micro-CT scanning and histological analysis. The animal study was approved by the Animal Care and Experiment Committee of Shanghai Jiao Tong University Affiliated Sixth People's Hospital and was conducted according to the National Research Council Guide for Care and Use of Laboratory Animals.

#### Micro-computed tomography evaluation

Femur samples were subjected to the micro-computed tomography (CT) (Laboratory Micro-CT Scanner Explore RS 80, GE Healthcare, Little Chalfont, UK) for morphological and structural evaluation. The X-ray tube was set at 80 kV and 450μA with a scan resolution of 45 μm and an exposure time of 400 ms. Bone radio morphometric analysis was performed using Micro View 2.2 Advanced Bone Analysis Application software (GE Health Systems, Waukesha, WI, USA). Femoral tunnel volume, bone-implant contact (BIC), bone mineral density (BMD),and the bone volume/total tissues volume (BV/TV) were analyzed as we previously reported [20].

#### Histology and immunofluorescence analysis

Formalin-fixed samples were decalcified in 9% formic acid for 9 weeks at room temperature, dehydrated with ethanol, and then embedded in paraffin. Hematoxylin–eosin (H–E) staining, Mallory staining and Safranin O/fast green staining were performed on 4-μm consecutive sections. The relative area of fibrocartilage tissue at the tendon–bone interface was calculated using Image-Pro Plus (Media Cybernetics, USA) accordingly. Furthermore, a modified histological score system for tendon–bone healing in ACL reconstruction was adopted for histological analysis of the tendon–bone interface [9].

Immunofluorescent staining for M1 or M2 macrophage marker was performed using anti-CD11b (1:500, Abcam, USA) and anti-Arg1 (1:200, Sigma-Aldrich, Germany). The stained slides were observed by fluorescence microscopy (Leica DM2500, Leica, Germany). Image Pro Plus 6.0 was used to quantify the positive staining area for each marker.

#### Western blot analysis

Tissues at the screw insertion site were collected at each time point and immediately frozen. The western blot analysis was consistent with previous protocol in Real time-polymerase chain reaction assay section.

### Statistical analysis

The results were expressed as numbers, medians, percentages and means ± SD. Statistical analysis was performed using the SPSS 27.0 software package (SPSS Inc., Chicago, USA). To compare data obtained from two groups, Student’s *t*-test was performed. *P*-values <0.05 were considered significant. For each assay, at least three independent experiments with duplication were conducted for averaged data.

## Results

### Cytocompatibility of HP Mg extracts on polarized macrophages

RAW 264.7 cells were treated with IFN-γ/LPS or IL-4/IL-10 to generate M1 or M2 macrophages, respectively. Induced M1 and M2 macrophages were treated with HP Mg extracts (M1-Mg, M2-Mg) or without HP Mg extracts (M1-Con, M2-Con) for 24 h. The Mg ion concentration of HP Mg extracts was 4.16 ± 0.21 mM, and the pH was 7.93 ± 0.16.

**Figure 2.**
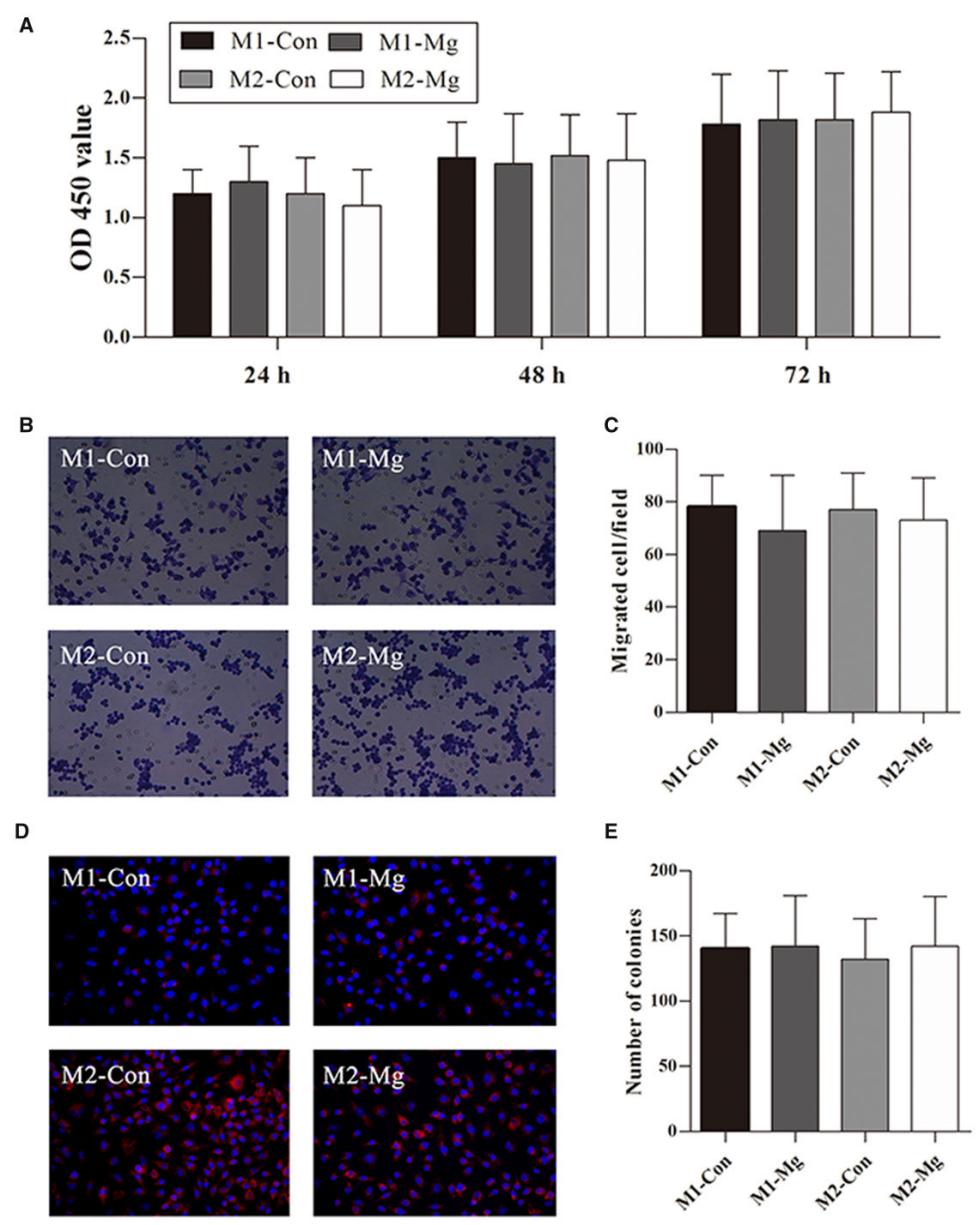
Cell viability (**A**), invasion ability (**B**, **C**) and colony formation ability (**D**, **E**) of the induced M1 macrophage group (M1-Con and M1-Mg) and induced M2 macrophage group (M2-Con and M2-Mg) after the treatment with (M1-Mg, M2-Mg) or without (M1-Con, M2-Con) HP Mg extracts.

Cell viability assay was performed to determine the cytotoxicity of HP Mg to macrophages. CCK-8 proliferation assay showed that HP Mg extracts did not affect cell proliferation of M1 or M2 macrophages, as no significant differences were observed between the two groups in OD 450 value at all the time points (Fig. 2A).

The invasion ability and the colony formation ability of cells were compared between control and HP Mg groups (M1-Con vs M1-Mg and M2-Con vs M2-Mg). The invasion and the colony formation phenotypes of macrophages were not altered by HP Mg in the medium post-treatment (Fig. 2B–E).

Together, these results indicated that HP Mg had good cytocompatibility to highly polarized macrophages without significant influence on cell viability, invasion ability or colony formation ability.

### Hp Mg extracts modulated M1/M2 macrophage polarization and related cytokines expression *in vitro*

**Figure 3.**
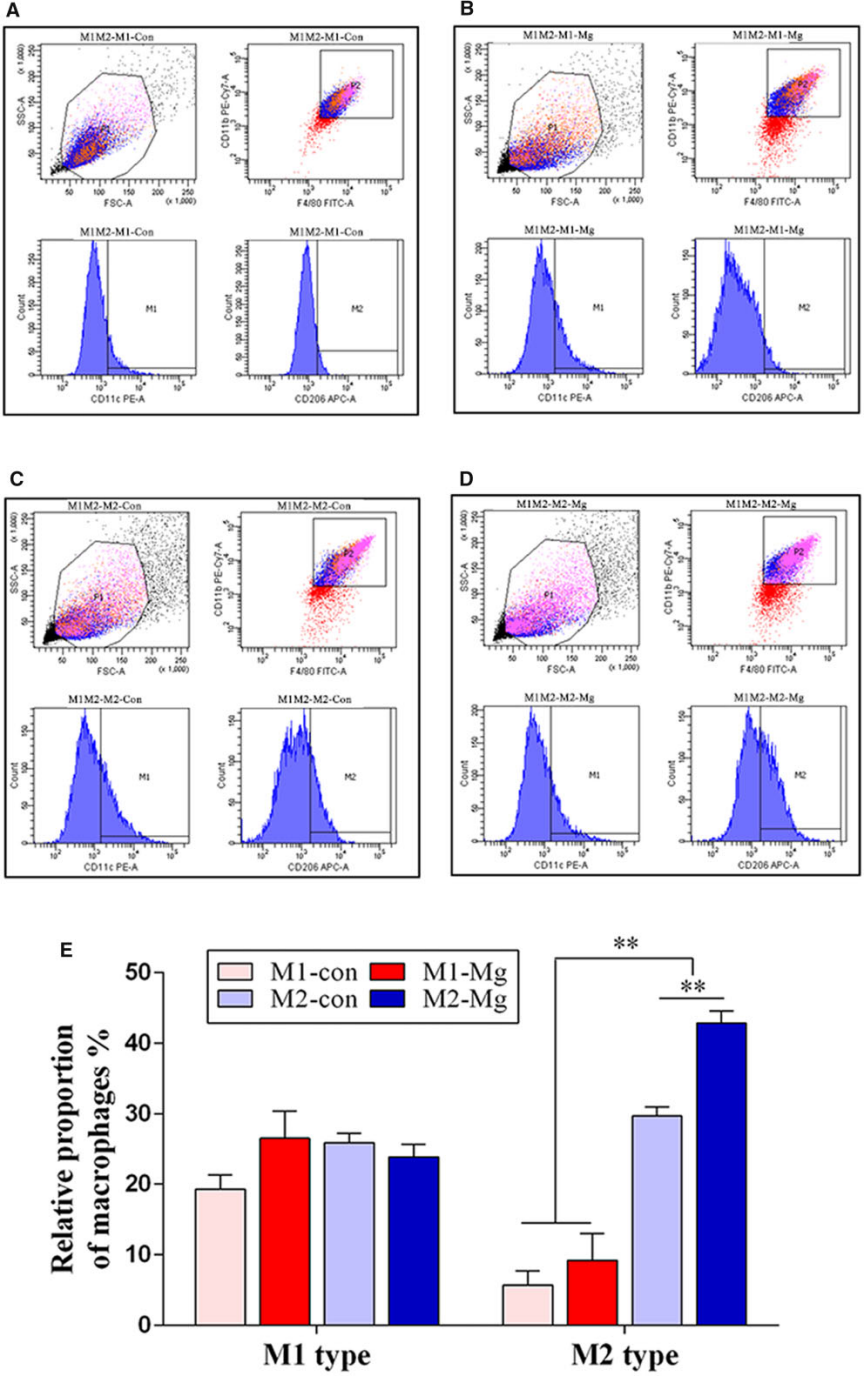
(**A**–**D**) Identification of M1 and M2 macrophages in the induced M1 groups and the induced M2 groups with or without HP Mg extracts treatment using flow cytometry. (**E**) The relative proportion of M1 macrophages and M2 macrophages in each group (M1-Con, M1-Mg, M2-Con and M2-Mg) (**P < 0.01).

Flow cytometry reflected the expression levels of M1 specific marker CD11c and M2 specific marker CD206. The relative proportion of polarized macrophages revealed the influence of the HP Mg extracts on macrophage polarization. As shown in Fig. 3, there is no significant difference between the M1-Con group and the M1-Mg group in the relative proportion of M1 macrophages (19.31 ± 2.01% vs 26.52 ± 3.80%, *P* > 0.05). No obvious differences were found between the M2-Con group and the M2-Mg group in the relative proportion of M1 macrophages (25.89 ± 1.27% vs 23.86 ± 1.74%, *P* > 0.05). Actually, the relative proportion of M2 macrophages obviously increased in the induced M2 groups (M2-Con group, M2-Mg group) compared with the induced M1 groups (M1-Con group, M1-Mg group) (*P* < 0.01). Furthermore, the relative proportion of M2 macrophages in the M2-Mg group was significantly higher than that in the M2-Con group (42.80 ± 1.74% vs 29.66 ± 1.27%, *P* < 0.01). The above results indicated that HP Mg extracts could promote the effects of M2 macrophage inducers on M2 polarization.

**Figure 4.**
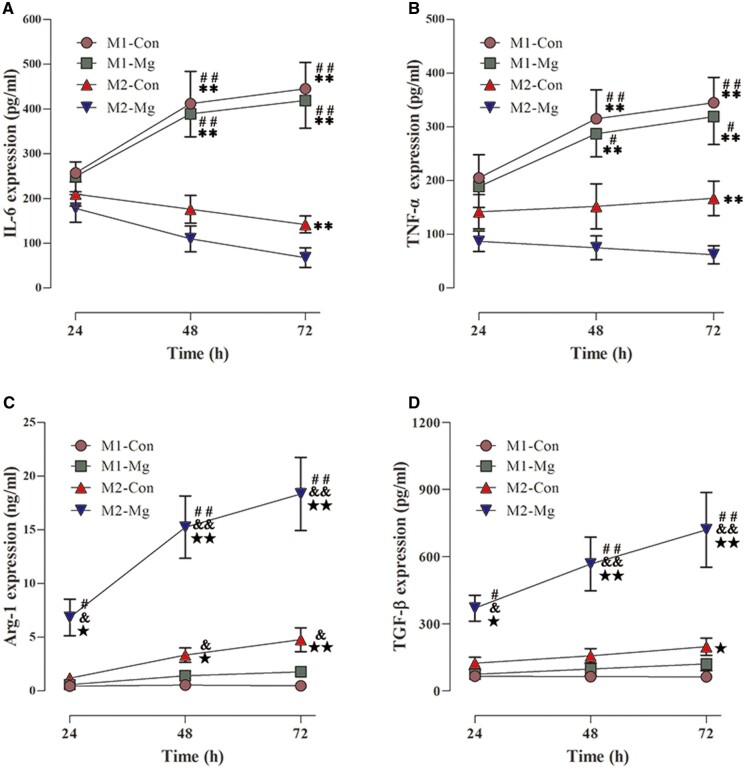
The expression of cytokines including IL-6 (**A**), TNF-α (**B**), Arg1 (**C**) and TGF- β (**D**) in the induced M1 groups and the induced M2 groups with or without HP Mg extracts treatment (*P < 0.05 compared with the M2-Mg group, **P < 0.01 compared with the M2-Mg group; ^#^*P* < 0.05 compared with the M2-Con group, ^##^*P* < 0.01 compared with the M2-Con group; ^&^*P* < 0.05 compared with the M1-Mg group, ^&&^*P* < 0.01 compared with the M1-Mg group; ^★^*P* < 0.05 compared with the M1-Con group, ^★★^*P* < 0.01 compared with the M1-Con group).

The expression of inflammatory cytokines (IL-6, TNF-α) and tissue repair cytokines (Arg1, TGF-β) were quantified by ELISA assay. As shown in Fig. 4, IL-6 and TNF-α levels increased over time and peaked at 72 h in the induced M1 groups (M1-Mg, M1-Con), while the expression of IL-6 and TNF-α in the induced M2 groups (M2-Mg, M2-Con) decreased over time. Furthermore, the expression levels of IL-6 and TNF-α were significantly higher in the induced M1 groups than in the induced M2 groups at 72 h (M1-Con vs M2-Con, and M1-Mg vs M2-Mg, *P* < 0.01). On the contrary, Arg1 and TGF-β levels gradually increased and reached a peak at 72 h in the induced M2 groups. The expressions of Arg1 were significantly higher in the induced M2 groups than in the induced M1 groups at 72 h, meanwhile, TGF-β expressions were relatively higher in the induced M2 groups than in the induced M1 groups at both 48 and 72 h. These results indicated that inflammatory cytokines could be upregulated by the M1 macrophage inducers, and tissue repair cytokines could be activated by the M2 macrophage inducers.

There were no significant differences in the IL-6 and TNF-α levels between the M1-Con and the M1-Mg group at all the time points (*P* > 0.05). However, IL-6 and TNF-α expressions in the M2-Mg group were significantly lower compared with the M2-Con group at 72 h (*P* < 0.05). Furthermore, the expression of Arg1 and TGF-β in the M2-Mg group was significantly higher than that in the M2-Con group at all the time points (*P* < 0.01). The results suggested that HP Mg inhibited the production of inflammatory cytokines and stimulated tissue repair cytokines in highly polarized M2 macrophages.

### Signaling pathway involved in M2 macrophage polarization modulated by HP Mg extracts

**Figure 5.**
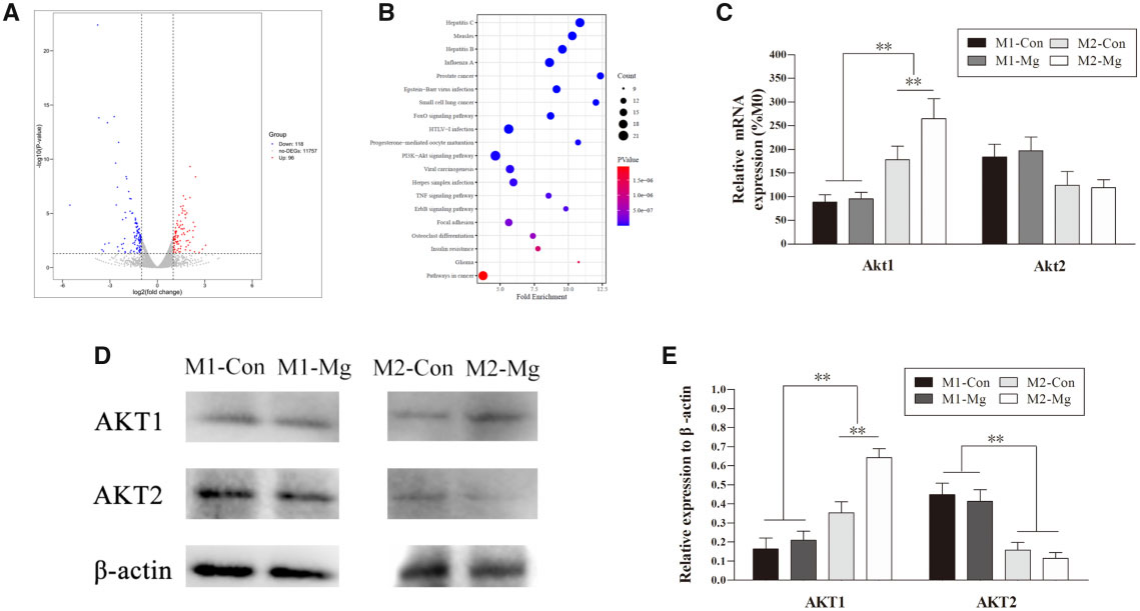
(**A**) Volcano plot of the differentially expressed genes (DEGs). Points on the right (red), middle (gray) and left part (blue) represent up-regulated, down-regulated and non-the DEGs. (**B**) Bubble chart referring to the top 20 enriched pathways in the KEGG. RT-PCR (**C**) and western blot (**D**) of AKT1 and AKT2 expression in the induced M1 and M2 groups with or without HP Mg extracts treatment. (**E**) Densitometry of AKT1 and AKT2 in the western blot assay (**P < 0.01).

To explore which signaling pathways dictate the effects of HP Mg on macrophage polarization, we conducted a statistical comparison of differentially expressed genes (DEGs) between the M2-Con and M2-Mg groups. Ninety-six upregulated genes and 118 downregulated genes were identified in the M2-Mg group compared with the M2-Con l group (Fig. 5A). Moreover, we evaluated DEGs by KEGG analysis and identified the top 20 KEGG pathways based on the most significant fold enrichment (Fig. 5B). Among these 20 KEGG pathways, DEGs between M2-Con and M2-Mg are most obviously enriched in the PI3K-AKT signaling.

We further performed RT-PCR to verify the RNA-seq results. As shown in Fig. 5C, the mRNA level of Akt1 in the induced M2 groups was significantly higher than that in the induced M1 groups (*P* < 0.01), and there was no difference in Akt1 mRNA level between the M1-Con group and the M1-Mg group. Notably, M2-Mg group showed a remarkably higher Akt1 mRNA level than M2-Con group (*P* < 0.05). As for the mRNA expression of Akt2, a higher but not significant trend existed in the induced M1 groups than the induced M2 counterparts, which was independent of HP Mg treatment. HP Mg treatment was found able to upregulate Akt1 transcript in M2 macrophages, which was in consistence with the RNA-seq result.

AKT1 and AKT2 protein levels in polarized macrophages with or without HP Mg extracts treatment were determined by western blots (Fig. 5D and E). The expression of AKT1 was upregulated in the induced M2 groups compared with the induced M1 groups (*P* < 0.01), and HP Mg extracts further promote AKT1 expression in M2 macrophages (*P* < 0.05). On the contrary, the AKT2 protein levels were higher in the induced M1 macrophages than in the induced M2 macrophages, but HP Mg extracts had no obvious influence on the AKT2 expression. Combining both transcriptional and translational patterns, it could be speculated that Mg could specifically modulate AKT1 expression in M2 macrophages without interfering with AKT2 signaling.

### Influence of polarized macrophages on fibrochondrocyte differentiation of hBMSCs with or without HP Mg extracts treatment

**Figure 6.**
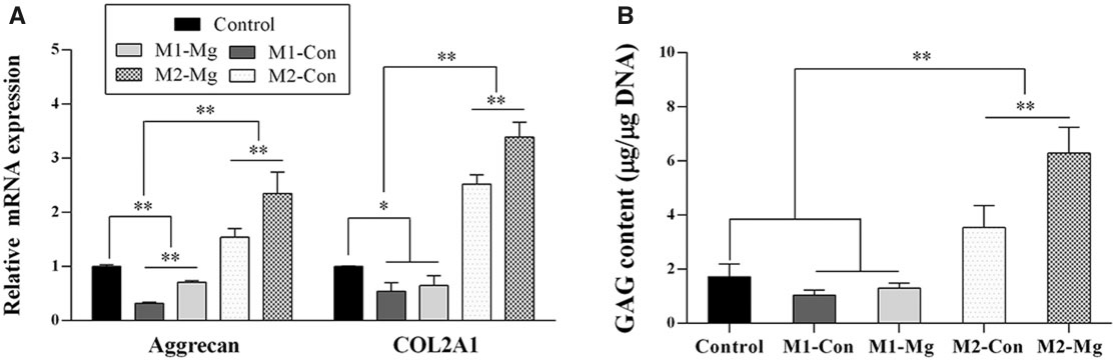
Gene expression of fibrochondrocyte markers (aggrecan, COL2A1) (**A**) and GAG content (**B**) of hBMSCs after co-culturing with CM harvested from M1-Con group, M1-Mg group, M2-Con group, M2-Mg group and control group (**P < 0.01).

The RT-PCR analysis in Fig. 6A showed the expression of fibrochondrocyte markers (Aggrecan and COL2A1) within hBMSCs after co-culturing with the polarized macrophages-CM. The induced M1 groups (M1-Con and M1-Mg) had significant lower Aggrecan and COL2A1 expression compared with the control group, whereas the induced M2 macrophage groups (M2-Con and M2-Mg) had obviously higher expression compared with both the induced M1 macrophage groups and the control group (*P* < 0.01). Furthermore, in the induced M1 and M2 macrophages, HP Mg treatment group had obviously higher expression of fibrochondrocyte markers than the control groups (M1-Mg group vs M1-Con group, and M2-Mg group vs M2-Con group, *P* < 0.01).

The GAG assay reflected the production of fibrocartilage matrix in each group. As in Fig. 6B, the GAG production in the M2-Mg group was obviously higher than any other groups (*P* < 0.01). The GAG production in the induced M2 groups (M2-Con and M2-Mg) were higher than the control group (*P* < 0.01). Although the GAG production decreased after co-culturing with the induced M1 groups, no obvious differences were detected (*P* > 0.05).

These results indicated that M2 macrophages promoted fibrochondrocyte differentiation of hBMSCs, while the M1 macrophages impeded the process. And HP Mg could enhance fibrochondrocyte differentiation modulated by the polarized macrophages.

### *In vivo* study of macrophage polarization in ACL reconstruction fixed by HP Mg screws

#### Functional recovery after ACL reconstruction

No signs of joint swelling, limping, or wound infection was noticed at the implantation site before sacrifice. All rabbits returned to normal activity without immobilization 1 week after ACL reconstruction. According to Supplementary Fig. S1, the postoperative knee ROM in both groups decreased at 2 weeks (92.6° ± 17.1° vs 87.7° ± 14.1°) and upswung at 4 weeks (118.4° ± 22.7° vs 128.0° ± 19.7°). Twelve weeks after the surgery, ROM scores of the HP Mg group and the Ti group were within the normal range and showed no significant difference when compared with each other.

#### The degradation behavior and mineral incorporation of HP Mg screws in ACL reconstruction

**Figure 7.**
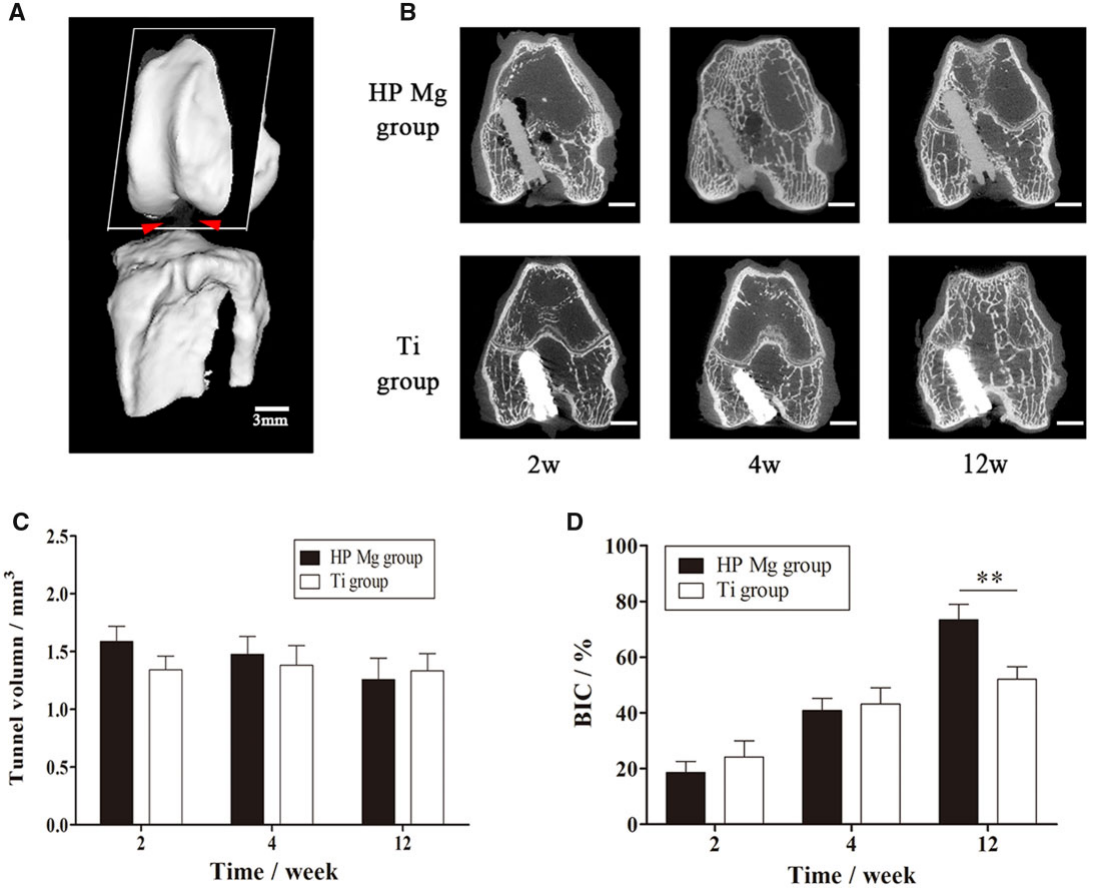
(**A**) Three-dimensional images of semitendinosus autograft fixed by the screws to the femoral tunnel in ACL reconstruction (scale bar means 3 mm). (**B**) Representative 2D micro-CT images of coronal sections of the femoral tunnel with HP Mg and Ti screws at 2, 4 and 12 weeks post-operatively (scale bar means 3 mm). (**C**) Volume of femoral tunnel in which the HP Mg screws inserted. (**D**) BIC calculated by micro-CT (**P < 0.01).

Representative 2D micro-CT images delineated the *in vivo* degradation behavior of HP Mg screws and *de novo* bone formation within the bone tunnel (Fig. 7A). At 2 weeks, periosteal reaction surrounding screws was active in both groups. At 4 weeks, bone tissues accumulated on the screw surface, especially at the entrance of the bone tunnel. The gas cavity surrounding the HP Mg screws was detected at 2 weeks and was absorbed at 4 weeks. Both the HP Mg screws and Ti screws were in direct contact with trabecular bones at 12 weeks (Fig. 7B). The tunnel volume of the HP Mg group gradually decreased from 1.59 ± 0.13 mm^3^ at 2 weeks, to 1.48 ± 0.14 mm^3^ at 4 weeks and 1.26 ± 0.13 mm^3^ at 12 weeks. Furthermore, there were no significant difference between the tunnel volume in the HP Mg group and the Ti group at each time point (Fig. 7C). The BIC presented in Fig. 7D showed the bone tissues surrounding the screws kept growing during the experimental period and there was significantly more bone ingrowth into the HP Mg screws than Ti screws at 12 weeks (73.4 ± 5.5% VS 52.1 ± 4.5%, *P* < 0.05). The tunnel volume and BIC results of the HP Mg group were consistent with its uniform corrosion behavior, indicating that the HP Mg screws provided prolonged fixation of tendon graft, which gradually transferred from the mechanical fixation into the biological fixation with the bony ingrowth during screw degradation.

**Figure 8.**
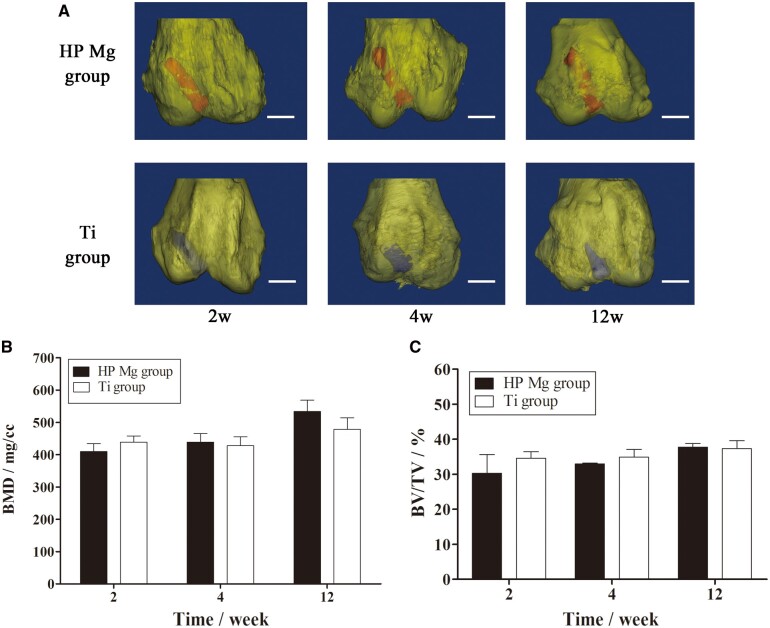
(**A**) Three-dimensional images of the distal femur (yellow) with the HP Mg (red) and Ti screws (blue) at 2, 4 and 12 weeks (scale bar means 3 mm). (**B**, **C**) BMD and BV/TV of the distal femur at 2,4 and 12 weeks postoperatively.

In the representative 3D micro-CT images of the femoral condyle (Fig. 8A), the HP Mg screws were in their original position without obvious displacement throughout the experimental period, indicating the firm fixation. The osseointegration of HP Mg and Ti screws obviously increased with time, which was consistent with the BIC data. Furthermore, The BMD and BV/TV of the distal femur were comparable between the HP Mg group and the Ti group at all the time points (Fig. 8B and C). These results collectively indicated that the mineral deposition on HP Mg screws during corrosion was compatible with the biological changes in the distal femur, which was responsible for the biological fixation of the tendon graft after ACL reconstruction.

#### Hp Mg screws promoted regeneration of fibrocartilaginous entheses in the ACL reconstruction

**Figure 9.**
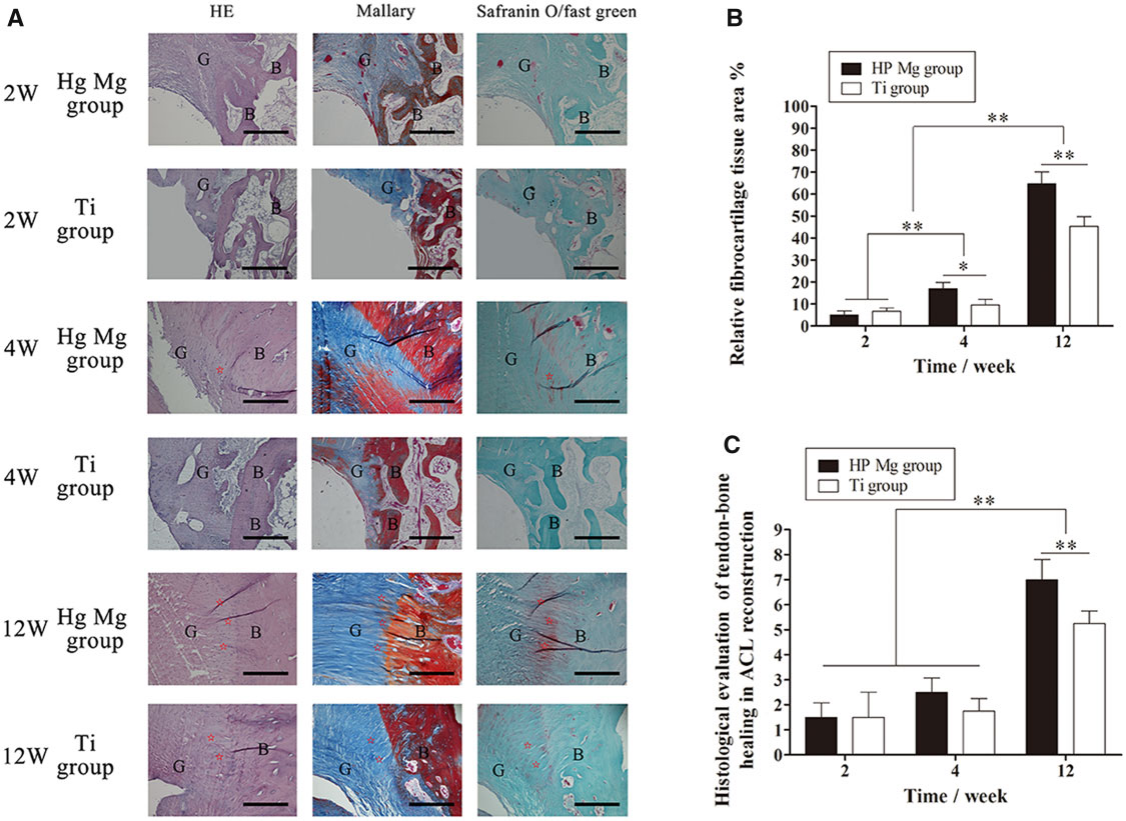
Histological analysis of the tendon–bone interface at 2, 4 and 12 weeks after surgery. (**A**) Representative HE, mallory and safranin O/fast green stained sections (G represents tendon graft, B represents bone, ⋆ represents fibrocartilage tissues, scale bar means 100 μm). (**B**) Histological scores of the tendon–bone interface in ACL reconstruction. (**C**) Calculated fibrocartilage area at the tendon–bone interface (**P < 0.01).

The reconstruction process of the tendon–bone interface was shown in Fig. 9A. In the early phase of ACL reconstruction, both the HP Mg and the Ti groups showed graft degradation and vascularization at the tendon–bone interface. The collagen bundles in the HP Mg group were regularly distributed within a relatively large area. In comparison, more splitting collagen defragment at the tendon–bone interface could be found in the Ti group.

At 4 weeks, the early regeneration of fibrocartilage tissues was observed at the tendon–bone interface. In the HP Mg group, fibrochondrocytes positively stained by Safranin O/fast green could be detected between the tendon graft and the bone tunnel, although the fibrocartilage layer was not obvious. The fibrocartilage tissues in the HP Mg group blended with fibrovascular tissues according to the Mallary staining. Meanwhile, the staining area of fibrocartilage tissues in the Ti group was obviously smaller than that in the HP Mg group (17.0 ± 2.8% VS 9.6 ± 2.5%, *P* < 0.05, Fig. 9B).

The highly differentiated fibrocartilage tissue was observed with a clear boundary at the surface of the insertion site 12 weeks after surgery. The HP Mg group had a fibrocartilage interlayer at the tendon–bone interface according to the Safranin O/fast green staining and Mallary staining. In comparison, the positively stained zone of fibrocartilage tissues in the Ti group was markedly smaller (64.6 ± 5.4% VS 45.3 ± 4.5%, *P* < 0.01, Fig. 8B). Furthermore, the histological score of the HP Mg group at the tendon–bone interface increased greatly and was significantly higher than that of the Ti group (*P* < 0.01, Fig. 9C). All the results indicated that the HP Mg screws promoted regeneration of fibrocartilaginous entheses in the process of tendon–bone healing.

#### The M1/M2 polarization behavior at the tendon–bone interface during the ACL reconstruction

To estimate the polarization of macrophages at the tendon–bone interface, immunofluorescent staining was employed for the CD11b (M1 maker) and Arg1 (M2 maker).

**Figure 10.**
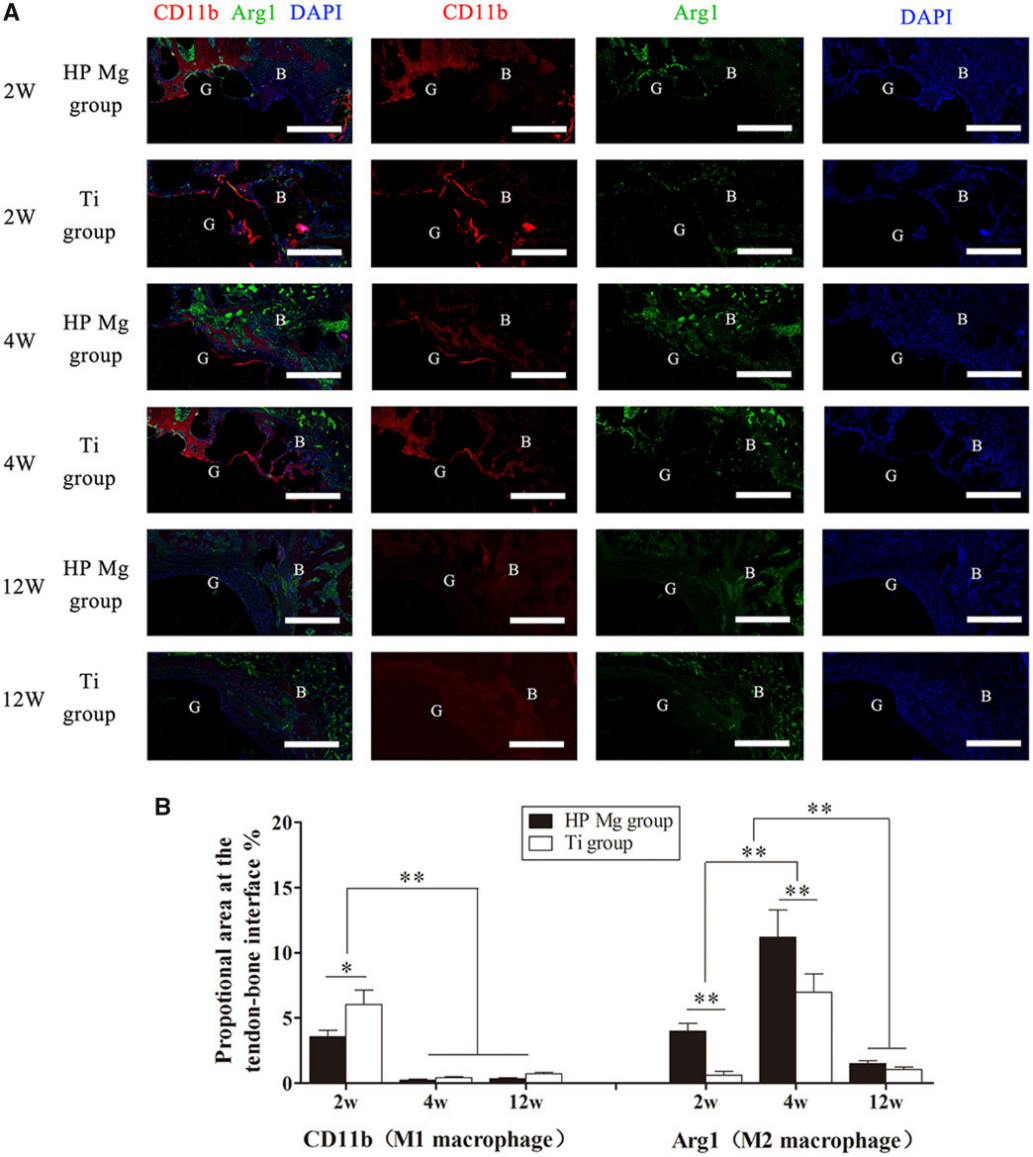
(**A**) Immunofluorescence showing colocalization of M1 (CD11b) and M2 (arg 1) macrophages at the tendon–bone interface of HP Mg group and Ti group at 2, 4 and 12 weeks after ACL reconstruction (scale bar means 150 μm). (**B**) Quantitation of macrophages expressing M1 and M2 markers determined by proportional area staining for CD11b (M1 macrophages) and Arg1 (M2 macrophages) at the tendon–bone interface (*P < 0.05, **P < 0.01).

As shown in Fig. 10, the expression of CD11b (red) at the tendon–bone interface was distinctly upregulated at 2 weeks and decreased at 4 weeks (*P* < 0.01). The expression of CD11b was higher in the Ti group than in the HP Mg group at 2 weeks (*P* < 0.05), while no significant difference existed between these two groups at 4 weeks. At 12 weeks, both groups showed no obvious expression of CD11b. It indicated that the M1 macrophage polarization reached the peak in the early phase of tendon–bone healing, and HP Mg screws might inhibit M1 macrophage polarization.

The expression of Arg1 (green) was more abundant in the HP Mg group than in the Ti group at 2 weeks (*P* < 0.01). Then the expression of Arg1 increased at 4 weeks, and it remained significantly higher in the HP Mg group than in the Ti group (*P* < 0.01). At 12 weeks, the expression of Arg1 gradually decreased and no significant differences were detected between these two groups. It indicated that the HP Mg screws might stimulate the accumulation of M2 macrophages at the tendon–bone interface, as well as switch the macrophage polarization from M1 to M2 at the early stage of ACL reconstruction.

#### The activation of AKT at the tendon–bone interface surrounding HP Mg screws

**Figure 11.**
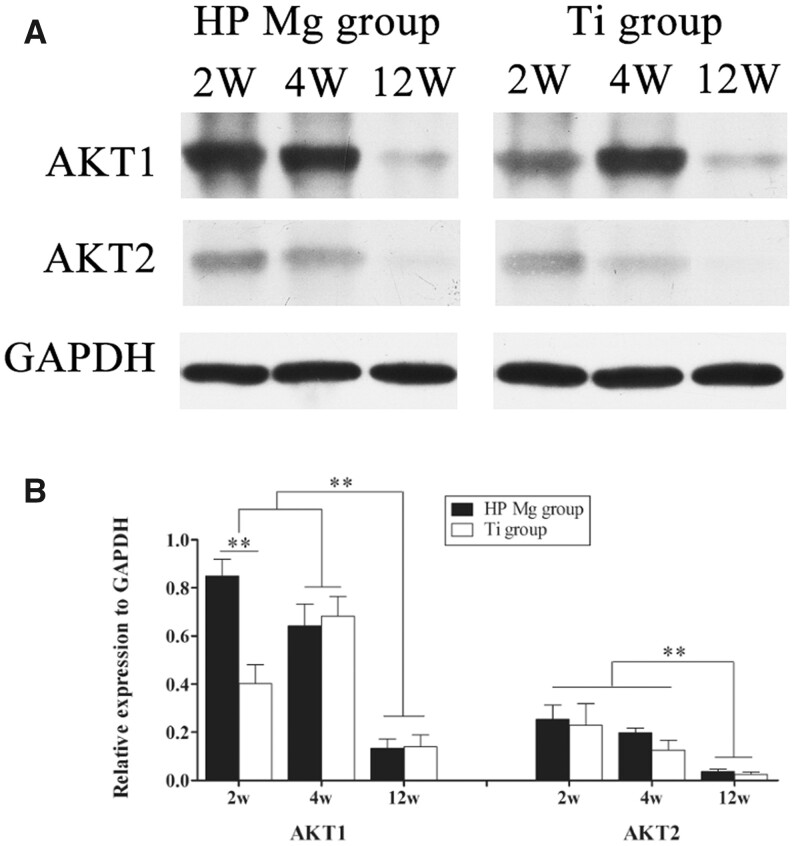
(**A**) Western Blot of AKT1 and AKT2 expression at the tendon–bone interface of HP Mg group and Ti group at 2, 4 and 12 weeks after ACL reconstruction. (**B**) Densitometry of AKT1 and AKT2 in the western blot assay (**P < 0.01).

The expression of AKT1 and AKT2 at the tendon–bone interface during ACL reconstruction was shown in Fig. 11. The AKT1 expression in the HP Mg group reached its peak at 2 weeks and remained at a high level at 4 weeks postoperatively without significant changes. On the contrary, the AKT1 expression in the Ti group was significantly lower than that in the HP Mg group at 2 weeks (*P* < 0.01). At 4 weeks, the AKT1 expression in the Ti group significantly increased and showed no significant differences compared with that in the HP Mg group. Then the expression of AKT1 in both groups greatly decreased at 12 weeks. Meanwhile, the expression of AKT2 in both the HP Mg group and the Ti group reached the peak at 2 weeks and then gradually decreased, while no significant differences were detected between the HP Mg group and the Ti group at each time point. These results suggested that the AKT1/AKT2 were activated in the early phase of tendon–bone healing, and the HP Mg screws could selectively promote the expression of AKT1 at the tendon–bone interface.

## Discussion

Our study reported the macrophage polarization at different phases of ACL reconstruction *in vivo* using a rabbit model and elucidated the further mechanisms both *in vitro* and *in vivo*. We demonstrated that Mg screws could switch the macrophage polarization from M1 to M2 at the early stage of ACL reconstruction. By further exploiting underlying mechanisms via RNA-Seq, we found the critical role of Mg degradation products on AKT1/2 signaling and subsequent effects on macrophage polarization and the stimulation of polarized macrophages on fibrochondrocyte differentiation. Importantly, our findings elucidated the mechanism of HP Mg screws on the promotion of tendon–bone healing during the ACL reconstruction in the view of immunomodulation and highlighted the potential application of Mg-based materials in clinical ACL reconstruction.

The effects of Mg screws on the tendon–bone healing process depend on numerous factors, including the implanted position, alloying component and degradation rate. Our study deeply elucidated the mechanism of intra-articular tendon–bone healing at the graft insertion site, by contrary to the intra-tunnel tendon–bone healing. The histological analysis in the present study showed that HP Mg group showed obvious fibrocartilage tissue formation at the intra-articular graft insertion site, rather than the intra-tunnel insertion site, at 12 weeks after surgery. It was widely accepted that once the surface healing was established at graft insertion site, the intra-tunnel healing was stress-shielded and the intra-tunnel tendon graft would be reabsorbed [23]. Moffat *et al.* also reported the distinct areas of fibrocartilage transition at the regenerated tendon–bone interface could surpass the native interface and significantly improved biomechanical load to failure and graft stiffness [24]. Hence, it is reasonable to presume that the Mg screws promoted the fibrocartilage transition zone formation at the intra-articular graft insertion site, rather than the intra-tunnel insertion site, which could directly influence not only the stability of the reconstructed knee joint but also the biomechanical properties of the biodegradable fixation itself. A recent study reported by Wang *et al*. found the early tendon graft reabsorption fixed by HP Mg screws and more mineralized matrix incorporation in bone tunnel and less peri-tunnel bone loss with tendon graft absorption [11]. The different findings could be partially explained with the intra-tunnel tendon–bone healing, compared with the intra-articular tendon–bone healing at the graft insertion site, and both of them synergistically contributed to the increased biomechanical strength.

The healing at the tendon–bone interface occurs most frequently by forming of fibrovascular scar tissues rather than reforming a native entheses with distinct areas of fibrocartilage transition [25, 26]. The fibrovascular scar interface lacked the integrity of the native fibrocartilaginous entheses, resulting in the mechanical and structural inferiority [27]. Kawamura et.al previously described that the macrophages contributed to the scar tissue interface in the early phase of tendon–bone healing [17]. Hays observed that liposomal clodronate could deplete macrophages in the rat ACL reconstruction model and observed remodeling fibrocartilage tissues in the bone tunnel surrounding tendon graft at 28 days and distinct fibrocartilage transition zone at 42 days, which were absent in the control group [16]. It was consistent with our present study that macrophages were remarkably reduced by HP Mg screws during the early inflammatory phase, leading to less formation of fibrovascular tissue. Consistently, the application of HP Mg screws showed integration with the surrounding cartilage in gross observation with rigid fixation at 9 and 12 weeks [9]. We believed that macrophages play critical roles in the regeneration of fibrocartilaginous entheses at the tendon–bone interface, and Mg-based biomaterials have potential in modulating the macrophages.

**Figure 12.**
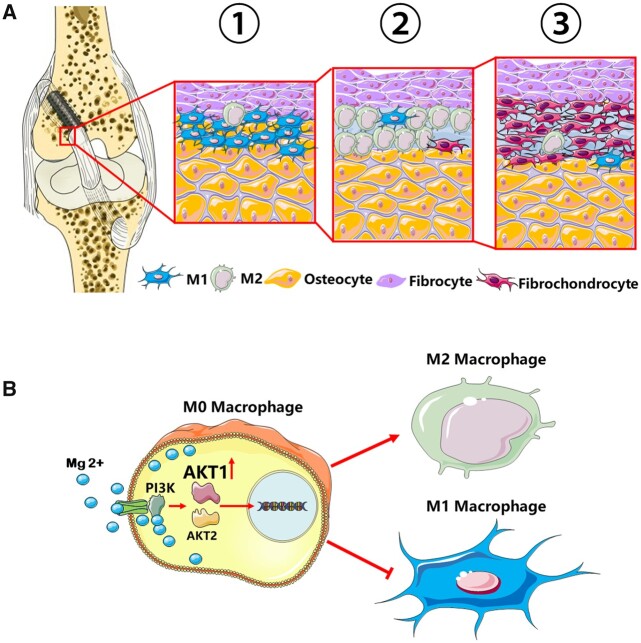
Schematic diagram showing the mechanism that Mg screws modulated macrophage polarization in the regeneration of fibrocartilaginous entheses during ACL reconstruction via AKT1/AKT2 alternative activation. (**A**) Tendon graft was fixed by the Mg screws in the femoral tunnel. At the entrance of the femoral tunnel, the surface healing process started with M1 macrophage polarization and accumulation at the tendon–bone interface in the early inflammation phase (Step 1). Macrophages then polarized to M2 type in the reparative phase (Step 2) and promoted the differentiation of BMSCs into fibrochondrocytes at the fibrocartilage interface (Step 3). (**B**) Implant-derived Mg^2+^ went through the ion channel and activated the PI3K-AKT signaling pathway. The AKT1, rather than AKT2, was selectively activated and resulted into the polarization of M2 macrophages in the reconstruction of ACL.

It is known that inflammatory M1 macrophages accumulate in the early inflammatory phase and have negative effects on tendon–bone healing, while M2 macrophages are necessary for inflammation resolution and matrix maturation in interface regeneration [28, 29]. It was proved in our *in vitro* co-culture model that the M1 macrophages impeded the fibrochondrocyte differentiation of BMSCs, while the M2 macrophages promoted the process of fibrochondrocyte differentiation. Actually, the wound-healing M2 macrophages were observed during the early stage of ACL reconstruction and reached the peak at 2 weeks postoperatively in our report. The results were in consistence with the pivotal roles of M2 macrophages in immunoregulation during ACL reconstruction [30]. During the inflammation resolution stage, M2 macrophages derived TGF-β could act directly on the immune cells and downregulate the majority of inflammatory cytokines [31]. The administration of TGF-β1 has been studied in animal models of ACL reconstruction and exhibited benefits in improving the load-to-failure, collagen fiber synthesis and *de novo* bone formation along the tunnel [32]. Besides TGF-β, various cytokines and growth factors including BMP-2 and VEGF have also been reported to enhance the graft healing process, and they were also largely derived from M2 macrophages [33]. Mg products could theoretically promote fibrocartilaginous entheses, rather than the fibrovascular scar interface, and switch the inflammatory response toward resolution in the process of tendon–bone healing via the modulation of M2 macrophage polarization (Fig. 12A).

Our present study elucidated that Mg degradation products could switch the macrophage polarization from M1 type to M2 type at the early stage of tendon–bone healing process, indicating the potential of Mg screws to downregulate inflammatory activities of macrophages without impairing their later regenerative activity. Previous studies found that Mg products had an effect on inhibit M1 polarization of macrophages [34]. It was believed that decreased M1 macrophages altered the balance of metalloproteinases (MMP) and their inhibitors (TIMPs), which could subsequently relieve inflammation and facilitate wound healing. We observed that HP Mg screws stimulated the accumulation of M2 macrophages at the tendon–bone interface 2 and 4 weeks after the ACL reconstruction, but the M1 macrophages were relatively less surrounding the HP Mg screws in the 2 weeks postoperatively. Considering that the influence of HP Mg extracts on M1 macrophage polarization was not obvious *in vitro*, Mg products might promote proliferation and accumulation of M2 macrophages without affecting M1 macrophages during the polarization. Li *et al*. investigated the M1/M2 polarization profile of macrophages cultured with the Mg-doped Ti, and detected lower levels of pro-inflammatory cytokines but higher levels of anti-inflammatory cytokines, compared with control cells [35]. However, Luthringer-Feyerabend cultured the U937 cells differentiated macrophages with the Mg-based extracts, and proved that both M1 and M2 profiles of macrophages were stimulated by pure magnesium [36]. Differences in cell culture system may account for the controversial findings. In the present study, we used M1/M2 macrophage inducers before treatment of HP Mg extracts. The findings supported our hypothesis that Mg screws could promote the M2 macrophages without M1 macrophages apoptosis in the macrophage polarization process.

To investigate the underlying mechanism by which Mg modulated macrophage polarization, we performed RNA-seq detection and WB assays, and found that AKT1 expression was highly upregulated in the Mg-induced M2 macrophages, while AKT2 expressions remained inapparent. AKTs family, also known as PKBs, serves as an important mediator in PI3K/AKT pathway. Previous studies have elucidated that PI3K/AKT activity was a crucial molecular mechanism underlying anti-inflammation process and macrophage polarization [37]. Of note, Jin *et al*. revealed that PI3K/AKT activity was important in the anti-inflammatory capacity of Mg [38]. Our study showed that AKT1 expression was increased by HP Mg screws at the early stage of tendon–bone healing. Meanwhile, HP Mg also inhibited M1 macrophages and upregulate M2 macrophages at the interface. Given that a previous study has implied that AKT1 was an important mediator to promote M2 macrophage polarization [39], AKT1 could be postulated to govern the anti-inflammatory capacity of Mg in the modulation of M2 macrophage polarization. Additionally, Liu *et al.* reported the coordination mode of Mg^2+^ in the stable conformation of AKT1 and revealed an indispensable role of Mg^2+^ in AKT1 activation [40]. Altogether, our study supported that the degradable products of HP Mg screws coordinated with the activated conformation of AKT1 and stimulated the transition of macrophages to M2 subtype via PI3K/AKT pathway, through which Mg^2+^ could inhibit inflammation and promoted tendon–bone healing during ACL reconstruction (Fig. 12B).

NF-κB signaling pathway has also been reported involved in Mg-modulated macrophage polarization. Sun *et al*. indicated that the micron-sized Mg particles (MgMPs) extracts could convert macrophages from M0 to M2 phenotype, which was consistent with our results [41]. The authors proposed the inhibition of NF-κB activation was critical in Mg modulated macrophage polarization. Mafalda Bessa-Gonc *et al.* reported that fibrinogen (Fg) and Mg combination biomaterials impaired macrophage M1 polarization and decreased NF-κB p65 phosphorylation, which was typically involved in inflammatory response [42]. They also mentioned that FgMg combination extracts inhibited M1-stimulated NF-κB p65 to a greater extent than those of individual materials. Activation of the PI3K-Akt pathway has been considered as a negative regulator of NF-κB signaling in macrophages, promoting anti-inflammatory responses [43]. Diaz-Guerra *et al*. conducted the nonspecific inhibition of PI3K signaling in TLR-activated cells, which led to NF-κB activation augmentation and M1 macrophage polarization [44]. Actually, we also observed that PI3K-AKT signaling was most obviously enriched in macrophage M2 polarization, which was promoted by the HP Mg screws, according to the RNA-seq assay, bio-informatic analysis and *in vivo* immunofluorescence and WB assay of rabbit ACL reconstruction model.

Although the biodegradable HP Mg screws have been proved as the potential modulator of macrophage polarization in the ACL reconstruction, some limits of the current study should still be realized. Firstly, the source of commercial primary antibody to rabbit models was limited, we alternatively used WB assays rather than immunofluorescence assay for the expression of AKT1/AKT2 in the rabbit model of ACL reconstruction. Secondly, as there were huge differences between *in vitro* and *in vivo* biological environments, it was still a challenging issue to predict the most suitable corrosion rate of Mg implants for the transition of polarized macrophages in human ACL reconstruction.

## Conclusion

The current study elucidated macrophage polarization during the process of tendon–bone healing in the rabbit ACL reconstruction model and suggested that Mg could modulate the M2 macrophage polarization and promoted the regeneration of fibrocartilage interface via the selective activation of AKT1.

1. We firstly reported tendon–bone healing process in the rabbit model from the perspective of immunomodulation and macrophage polarization. It supported the further application of immunomodulation agents in the clinical treatment of ACL reconstruction.
2. The tendon-graft healing surrounding HP Mg screws at the graft insertion site was characterized by the formation of fibrocartilage transition zones at the tendon–bone interface. HP Mg screws hindered the fibrovascular scar interface and promoted regeneration of fibrocartilage tissues via modulation of macrophage polarization, which highlighted the potential applications of Mg interference screws as immunomodulatory implants.
3. In the further mechanism study, we found that implant-derived Mg^2+^ selectively activated AKT1 in the PI3K-AKT signaling pathway, and its potential target could activate the polarization of M2 macrophages in the reconstruction of ACL. It was regarded as the foundation of Mg interference screws in clinical trials

## Supplementary data

Supplementary data are available at *REGBIO* online.

## Supplementary Material

rbac067_Supplementary_Data
