# Supplementary material for: High-purity magnesium screws modulate macrophage polarization during the tendon–bone healing process in the anterior cruciate ligament reconstruction rabbit model
Source: Regen Biomater. 2022 Oct 10;9:rbac067. doi: 10.1093/rb/rbac067 (PMC9580517; doi:10.1093/rb/rbac067)
Supplement: rbac067_Supplementary_Data [file rbac067_supplementary_data.docx]

**Table 1. Primers for the RAW 264.7 and hBMSC in the RT-qPCR analysis**

| Name | Primer | Sequence |
| --- | --- | --- |
| GAPDH  (RAW 264.7) | Forward | 5‘- ATGGGTGTGAACCACGAGA -3’ |
|  | Reverse | 5‘- CAGGGATGATGTTCTGGGCA -3’ |
| Akt1  (RAW 264.7) | Forward | 5‘- AATGCACGGCGATTACACTC-3’ |
|  | Reverse | 5‘- GGACACTGGGTAGAGCAACT-3’ |
| Akt2  (RAW 264.7) | Forward | 5‘- CTGCCCTTCTACAACCAGGA-3’ |
|  | Reverse | 5‘-CATACACATCCTGCCACACG -3’ |
| 18s  (hBMSCs) | Forward | 5‘-GTTCTTAGTTGGTGGAGCGATTT-3’ |
|  | Reverse | 5‘-CGGACATCTAAGGGCATCACA-3’ |
| Aggrecan  (hBMSC) | Forward | 5‘- GGCTGCTGTCCCCGTAGAAGA-3’ |
|  | Reverse | 5‘- GGGAGGCCAAGTAGGAAGGAT-3’ |
| COL2A1  (hBMSC) | Forward | 5‘-GCTCCCAGAACATCACCTACC-3’ |
|  | Reverse | 5‘-TGAACCTGCTATTG CCCTCT-3’ |
